# Decomposition of educational differences in life expectancy by age and causes of death among South Korean adults

**DOI:** 10.1186/1471-2458-14-560

**Published:** 2014-06-05

**Authors:** Kyunghee Jung-Choi, Young-Ho Khang, Hong-Jun Cho, Sung-Cheol Yun

**Affiliations:** 1 Department of Preventive Medicine, Ewha Womans University School of Medicine, Seoul, Korea; 2 Department of Health Policy and Management & Institute of Health Policy and Management, Seoul National University College of Medicine, Seoul, Korea; 3 Department of Family Medicine, Asan Medical Center, University of Ulsan College of Medicine, Seoul, Korea; 4 Department of Clinical Epidemiology and Biostatistics, Asan Medical Center, University of Ulsan College of Medicine, 388-1 Pungnap2-dong, Songpa-gu, Seoul 138-736, Korea

**Keywords:** Life expectancy, Socioeconomic inequality, Socioeconomic factors, Health status disparities, Contribution

## Abstract

**Background:**

Decomposition of socioeconomic inequalities in life expectancy by ages and causes allow us to better understand the nature of socioeconomic mortality inequalities and to suggest priority areas for policy and intervention. This study aimed to quantify age- and cause-specific contributions to socioeconomic differences in life expectancy at age 25 by educational level among South Korean adult men and women.

**Methods:**

We used National Death Registration records in 2005 (129,940 men and 106,188 women) and national census data in 2005 (15, 215, 523 men and 16,077,137 women aged 25 and over). Educational attainment as the indicator of socioeconomic position was categorized into elementary school graduation or less, middle or high school graduation, and college graduation or higher. Differences in life expectancy at age 25 by educational level were estimated by age- and cause-specific mortality differences using Arriaga’s decomposition method.

**Results:**

Differences in life expectancy at age 25 between college or higher education and elementary or less education were 16.23 years in men and 7.69 years in women. Young adult groups aged 35–49 in men and aged 25–39 in women contributed substantially to the differences between college or higher education and elementary or less education in life expectancy. Suicide and liver disease were the most important causes of death contributing to the differences in life expectancy in young adult groups. For older age groups, cerebrovascular disease and lung cancer were important to explain educational differential in life expectancy at 25–29 between college or higher education and middle or higher education.

**Conclusions:**

The contribution of the causes of death to socioeconomic inequality in life expectancy at age 25 in South Korea varied by age groups and differed by educational comparisons. The age specific contributions for different causes of death to life expectancy inequalities by educational attainment should be taken into account in establishing effective policy strategies to reduce socioeconomic inequalities in life expectancy.

## Background

Investigation into contributions by specific causes and age groups to absolute socioeconomic inequalities in total mortality is important to understand mechanisms of socioeconomic health inequalities and to establish policies and intervention programs to reduce socioeconomic inequalities in health. Many studies have reported the contribution of causes of death in specific age groups to socioeconomic mortality inequalities in Asia as well as in western countries [1-5]. They revealed that the pattern of the contribution by specific causes of death varied by countries, which informs of different policy priorities for different countries.

Life expectancy is the expected years of life of a person remaining at a given age and a summary measure for mortality determined by the probability of death at each age [6]. It has important strengths in that it can be more easily understood than the age-standardized mortality rates by the public and compared between countries or changes over time [7-9]. In addition, life expectancy can be decomposed by death causes and specific age groups, which allows us to better understand mechanisms of socioeconomic inequalities in mortality.

Decomposition of socioeconomic inequalities in life expectancy by ages or causes has been mainly performed in western countries [10-12]. Some studies showed age-specific contributions to socioeconomic inequalities in life expectancy over time [7,10,13,14] while other studies reported patterns of cause-specific contributions [6,11-14]. However, there is still a paucity of studies investigating age and death cause contributions socioeconomic difference in life expectancy by socioeconomic position (SEP) with use of national data covering whole population. This study aimed to quantify age- and cause-specific contributions to socioeconomic differences in life expectancy at age 25 by educational level among adult men and women in South Korea (hereafter ‘Korea’) to provide evidence guiding intervention priorities.

## Methods

### Study subjects

We used national death certificate and census data in 2005 from Statistics Korea. The number of total deaths aged 25 and over was 239,166 in 2005. After excluding data without any information on level of education, causes of death or age being missing or inaccurate, the present study included 236,128 deaths (98.7% of total deaths, 129,940 men and 106,188 women). In 2005 national census, 15, 215, 523 men and 16,077,137 women aged 25 and over were identified and included in this study.

By law, all deaths must be reported to Statistics Korea within a month of their occurrence in Korea. Death registration in Korea is known to be complete for deaths occurring among those aged 1+ years since the mid-1980s [15]. Death certification by a physician was suggested as a very important factor to improve accuracy in reporting causes of death in Korea [16,17]. The proportion of death certified by physicians was 86.9% in 2005. The reliability of the educational level in death certificate data was reported to be substantial [18].

This study was approved by the Asan Medical Center Institutional Review Board, Seoul, Korea.

### Socioeconomic position (SEP) indicator

A level of own education was used as the SEP indicator of this study. Educational attainment was categorized into elementary school graduation or less, middle or high school graduation, or college graduation or higher. Elementary school and high school in Korea correspond to the International Standard Classification of Education (ISCED) 1 and ISCED 3, respectively whereas there is no schooling system in Korea relevant to ISCED 4 [19]. College is classified as ISCED 5.

Educational achievement among Korean population during the past decades has been remarkable along with the huge economic development. The enrollment rate in elementary school was 69.8% in 1951 but reached to 97.7% in 1980 and 98.6% in 2012 [20]. An explosive increase was observed for the enrollment rate in college or higher education skyrocketing from 4.2% in 1965 to over 60% in 2005. Thus, a very different educational distribution with age groups can be found in Korea. For example, 61.5% of women at age 25–29 years are classified into college of higher graduation while 76.1% of women at age 60–64 years are classified into elementary school graduation or less in 2005 [21].

### Statistical analysis

For life expectancy at age 25, life tables were constructed using 5- year probabilities of death by educational level. 5-year probabilities of death were calculated based on the age-specific death rates which were estimated from the number of death in death certificate data and the number of population in census data by age and educational level. Differences in life expectancy at age 25 by educational level were calculated.

Age- and cause-specific contributions to the educational differences in life expectancy at age 25 were estimated using Arriaga’s decomposition method [22]. The Arriaga method which has been widely used to decompose differences in life expectancy concerns a direct effect, an indirect effect, and an interaction effect of mortality difference on life expectancy. The direct effect reflects a consequence of a mortality difference in that age group. The indirect effect is due to a change in the number of survivors at the end of that age interval from a mortality change within a specific age group. The interaction effect results from the combination of the changed number of survivors at the end of the age interval and the lower (or higher) mortality rates at older ages. The total contribution of each age group to the change in life expectancy can be calculated by adding the direct, indirect and interaction effect [22,23]. By Arriaga’s decomposition method, the difference in life expectancy can be decomposed into ages and causes of death which enable us to explain life expectancy differentials in terms of the contribution of each factor. Higher mortality rate in low SEP than high SEP makes a positive contribution to socioeconomic differences in life expectancy. In other words, a positive contribution refers a contribution to the increase in educational differentials in life expectancy. The total life expectancy differential by SEP is the sum of the number of years attributed negatively or positively by deaths in each age group or cause.

Life expectancy was calculated by causes of death. A total of 8 broad and 17 specific (15 for men and 14 for women) causes of death were selected based on the main causes of death in South Korea [24] (see Table 1). Causes of death were coded using the 10^th^ version of the International Classification of Disease (ICD-10).

**Table 1:** Crude death rates (/100,000) by main causes of death in 2005 among Korean men and women

	**Total**	**Men**	**Women**
All causes	504.3	554.7	453.5
Infectious and parasitic diseases (A00-B99)	11.4	14.1	8.8
Respiratory tuberculosis (A15-A16)	5.5	7.8	3.3
All Cancers (C00-D48)	136.0	171.0	100.9
Stomach cancer (C16)	22.6	29.4	15.7
Liver cancer (C22)	22.5	33.8	11.2
Lung/bronchial cancer (C33-C34)	28.4	41.6	15.0
Endocrine, nutritional & metabolic diseases (E00-E88)	25.5	25.8	25.3
Diabetes mellitus (E10-E14)	24.2	24.4	24.0
Disease of the circulatory system (I00-I99)	116.2	112.1	120.4
Hypertensive disease (I10-I13)	9.3	6.6	12.1
Heart disease (I20-I51)	39.6	41.0	38.2
Cerebrovascular disease (I60-I69)	64.3	61.2	67.3
Diseases of the respiratory system (J00-J98)	29.4	34.6	24.1
Pneumonia (J12-J18)	8.6	9.0	8.2
Chronic lower respiratory disease (J40-J47)	15.5	18.9	12.2
Diseases of the digestive system (K00-K92)	23.1	33.4	12.8
Liver diseases (K70-K76)	17.3	27.5	7.1
All external causes (V01-Y89)	63.6	86.2	40.8
Transport accidents (V01-V99)	16.3	24.0	8.6
Suicide (X60-X84)	26.1	34.9	17.3

Data source: Statistics Korea. **The Causes of Death Statistics in 2005**.

## Results

**Figure 1:**
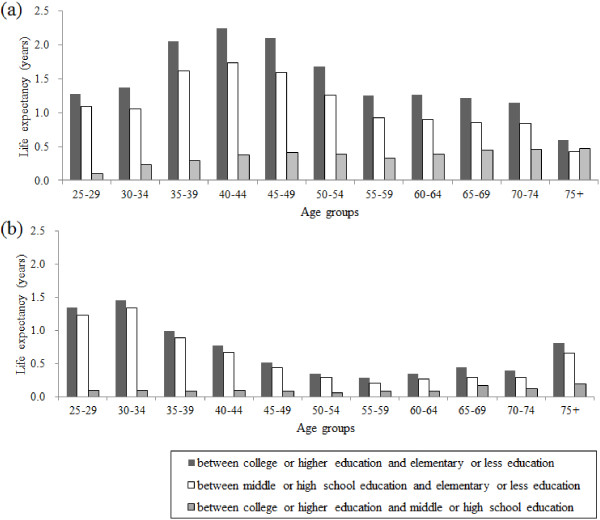
Age-specific contributions to the educational gaps in life expectancy at age 25 among Korean (a) men and (b) women.

Table 2 shows numbers of subjects and deaths and life expectancy at age 25 according to educational level. Middle or high school graduates accounted for about half of total subjects among both men and women (50.3% for men and 52.1% for women) while the numbers of deaths were the greatest among those with elementary school graduation or less (41.9% for men and 57.6% for women). Life expectancy at age 25 was 48.39 years in men and 54.75 years in women, respectively. Life expectancy stepwisely increased with education levels. Differences in life expectancy at age 25 between college or higher education and elementary or less education were 16.23 years in men and 7.69 years in women.Figure 1 shows age-specific contributions to the educational gap in life expectancy among Korean men and women. In men, those aged 40–44 as a single age group contributed most (13.9%) to educational differences in life expectancy at age 25 between college or higher education and elementary or less education. Contributions of ages between 35 and 49 to the educational differences in life expectancy were greater than those of ages other age groups. This was true for the educational differences between college or higher education and elementary or less education and true for the differences between middle or high school and elementary or less education. Meanwhile, older age groups aged 60–64 and over contributed significantly to the educational differences in life expectancy between middle or high school and college or higher education.Figure 1 also presents age-specific contributions in Korean adult women. Among women, younger age groups between 25 and 39 showed greater contributions than other older age groups. This was true for educational differences between college or higher education and elementary or less education and true for between middle or high school and elementary or less education. Meanwhile, older age groups aged over 65 contributed significantly to the educational differentials in life expectancy.

**Table 2:** Numbers of subjects and deaths, and life expectancy at age 25 by education levels among Korean men and women

**Education**	**No. of subjects**	**No. of deaths**	**Life expectancy at age 25**	**Life expectancy difference**
Men				
Total	15215523	129940	48.390	
Elementary or less	1862582	63705	36.189	16.226
Middle or high school	7653323	51562	48.503	3.912
College or higher	5699618	14673	52.415	Reference
Women				
Total	16077137	106188	54.754	
Elementary or less	4105545	87583	48.891	7.692
Middle or high school	7927474	15823	55.453	1.130
College or higher	4044118	2782	56.583	Reference

Table 3 presents cause-specific contributions to the life expectancy gap by education in Korean men. In broad causes of deaths, the contributions by cancers were greater than those of cardiovascular diseases in men while in women the contributions by cardiovascular diseases surpassed the contributions by cancers. This pattern was true for all the comparisons between educational levels considered. In both men and women, the contributions by external causes were significant, substantially accounting for total educational differences in life expectancy (about 28-29% in men and 20-24% in women).

**Table 3:** Cause-specific contributions (years and%) to life expectancy gap by education among Korean men and women

**Cause of death**	**Men**			**Women**		
	**I vs. III**	**I vs. II**	**II vs. III**	**I vs. III**	**I vs. II**	**II vs. III**
All causes	16.226 (100.0)	3.912 (100.0)	12.314 (100.0)	7.692 (100.0)	1.130 (100.0)	6.562 (100.0)
Infectious and parasitic diseases (A00-B99)	0.790 (4.87)	0.144 (3.69)	0.655 (5.32)	0.233 (3.03)	0.033 (2.88)	0.198 (3.02)
Tuberculosis (A15-A19)	0.504 (3.10)	0.097 (2.49)	0.423 (3.44)	0.126 (1.63)	0.023 (2.02)	0.100 (1.52)
Other infectious diseases	0.286 (1.76)	0.047 (1.20)	0.232 (1.88)	0.108 (1.40)	0.010 (0.86)	0.098 (1.50)
All Cancers (C00-D48)	2.799 (17.25)	0.857 (21.91)	1.945 (15.80)	1.041 (13.54)	0.113 (10.04)	0.921 (14.03)
Stomach cancer (C16)	0.529 (3.26)	0.117 (2.98)	0.493 (4.01)	0.183 (2.38)	0.025 (2.20)	0.155 (2.36)
Colorectal cancer (C18-C21)	0.017 (0.10)	0.001 (0.02)	−0.010 (−0.08)	0.062 (0.81)	−0.001 (−0.11)	0.064 (0.98)
Liver cancer (C22)	0.669 (4.12)	0.188 (4.81)	0.441 (3.58)	0.179 (2.33)	0.025 (2.17)	0.155 (2.36)
Lung/bronchial cancer (C33-C34)	0.928 (5.72)	0.334 (8.54)	0.677 (5.50)	0.179 (2.33)	0.052 (4.61)	0.123 (1.87)
Breast cancer (C50)	-	-	-	0.058 (0.75)	−0.015 (−1.32)	0.072 (1.10)
Uterine cervix/unspecified uterine cancer (C53-C55)	-	-	-	0.108 (1.40)	0.023 (2.04)	0.084 (1.28)
Prostate cancer (C61)	−0.028 (−0.17)	−0.033 (−0.83)	−0.026 (−0.21)	-	-	-
Other cancers	0.685 (4.22)	0.250 (6.40)	0.371 (3.01)	0.272 (3.54)	0.005 (0.45)	0.267 (4.08)
Endocrine, nutritional & metabolic diseases (E00-E88)	0.554 (3.42)	0.154 (3.94)	0.313 (2.54)	0.376 (4.88)	0.113 (10.03)	0.248 (3.77)
Diabetes mellitus (E10-E14)	0.474 (2.92)	0.138 (3.52)	0.243 (1.97)	0.337 (4.38)	0.106 (9.41)	0.217 (3.30)
Other endocrine diseases	0.080 (0.49)	0.017 (0.42)	0.056 (0.45)	0.039 (0.50)	0.007 (0.62)	0.028 (0.43)
Disease of the circulatory system (I00-I99)	1.949 (12.01)	0.612 (15.65)	1.167 (9.48)	1.624 (21.11)	0.328 (29.01)	1.287 (19.62)
Hypertensive disease (I10-I13)	0.068 (0.42)	0.039 (1.00)	0.044 (0.36)	0.109 (1.41)	0.040 (3.52)	0.066 (1.01)
Ischaemic heart disease (I20-I25)	0.318 (1.96)	0.062 (1.57)	0.156 (1.27)	0.273 (3.55)	0.065 (5.72)	0.206 (3.13)
Cerebrovascular disease (I60-I69)	1.117 (6.88)	0.416 (10.63)	0.661 (5.37)	0.801 (10.41)	0.168 (14.84)	0.640 (9.75)
Other cardiovascular diseases	0.447 (2.75)	0.096 (2.45)	0.306 (2.48)	0.441 (5.74)	0.056 (4.93)	0.376 (5.73)
Diseases of the respiratory system (J00-J98)	0.833 (5.13)	0.212 (5.43)	0.809 (6.57)	0.329 (4.28)	0.078 (6.87)	0.252 (3.85)
Pneumonia (J12-J18)	0.191 (1.18)	0.030 (0.76)	0.172 (1.40)	0.095 (1.23)	0.015 (1.36)	0.079 (1.20)
Chronic lower respiratory disease (J40-J47)	0.445 (2.74)	0.155 (3.96)	0.418 (3.39)	0.163 (2.12)	0.036 (3.17)	0.133 (2.03)
Other respiratory diseases	0.196 (1.21)	0.028 (0.71)	0.219 (1.78)	0.071 (0.92)	0.027 (2.35)	0.041 (0.62)
Diseases of the digestive system (K00-K92)	2.110 (13.00)	0.408 (10.44)	1.684 (13.68)	0.479 (6.23)	0.091 (8.01)	0.377 (5.75)
Liver diseases (K70-K76)	1.891 (11.65)	0.361 (9.22)	1.505 (12.22)	0.349 (4.54)	0.067 (5.91)	0.274 (4.18)
Other digestive diseases	0.219 (1.35)	0.048 (1.22)	0.179 (1.46)	0.130 (1.69)	0.024 (2.10)	0.103 (1.57)
All external causes (V01-Y89)	4.665 (28.75)	1.099 (28.09)	3.509 (28.50)	1.574 (20.46)	0.271 (24.01)	1.296 (19.75)
Transport accidents (V01-V99)	1.197 (7.38)	0.317 (8.09)	0.866 (7.03)	0.370 (4.81)	0.052 (4.64)	0.319 (4.86)
Suicide (X60-X84)	1.750 (10.79)	0.444 (11.36)	1.297 (10.53)	0.666 (8.66)	0.143 (12.68)	0.519 (7.90)
Other external causes	1.717 (10.58)	0.338 (8.64)	1.347 (10.94)	0.538 (6.99)	0.076 (6.69)	0.458 (6.99)
Ill-defined (R00-R99)	1.149 (7.08)	0.304 (7.77)	1.115 (9.06)	0.854 (11.10)	0.046 (4.09)	0.866 (13.20)
Residual	1.377 (8.49)	0.120 (3.07)	1.116 (9.06)	1.183 (15.37)	0.057 (5.06)	1.116 (17.01)

Note: I = College or higher, II = Middle or high school, III = Elementary or less.

**Figure 2:**
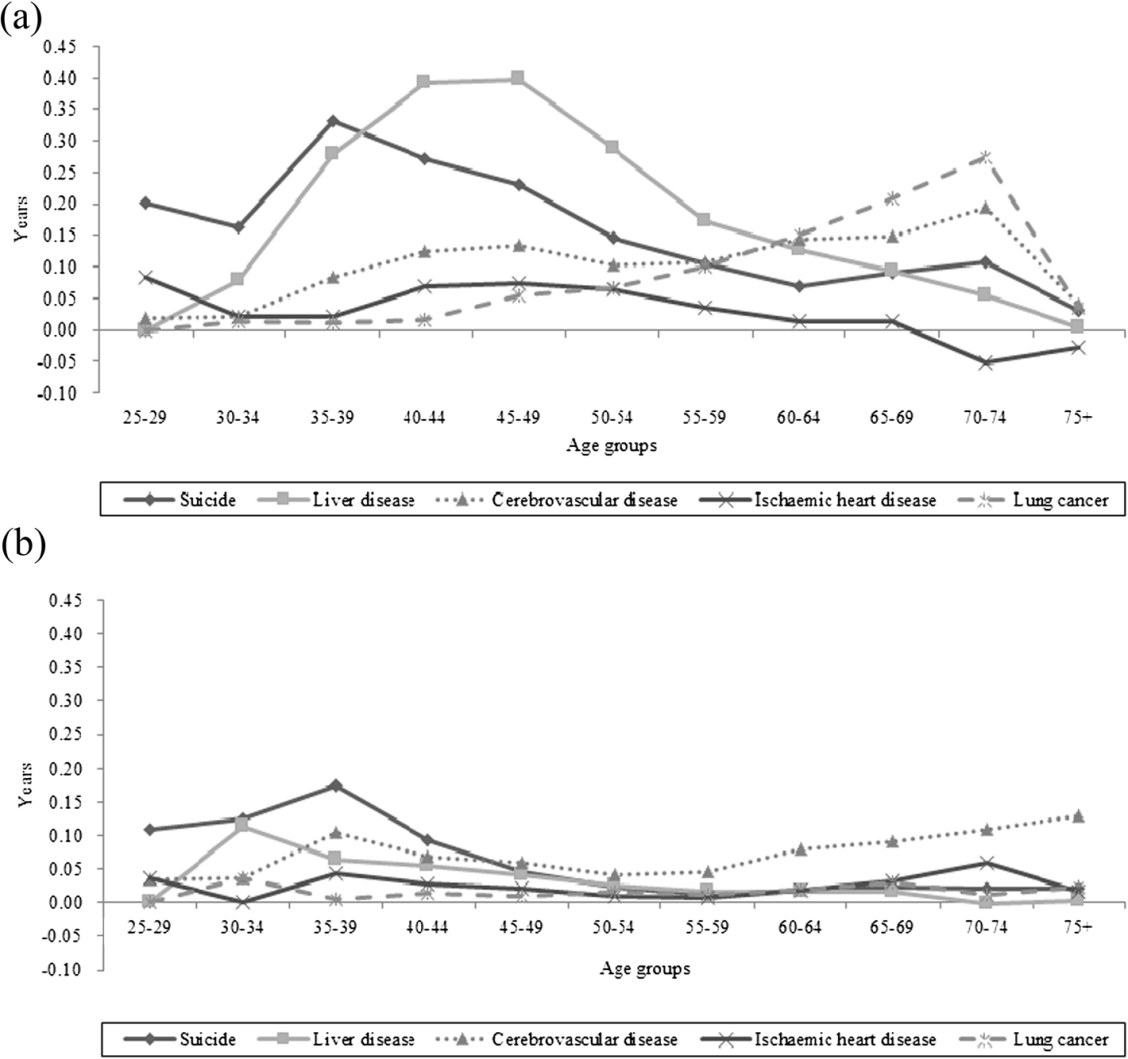
Age- and cause-specific contributions by major causes of deaths to educational differences in life expectancy at age 25 between college or higher education and elementary or less education among Korean (a) men and (b) women.

Table 3 also shows contributions by specific causes. Liver disease, suicide, transport accident, cerebrovascular disease, and lung cancer played important roles in explaining educational differences in life expectancy in men. Especially, the most important contribution among specific causes was made by liver disease, explaining about 9-12% of total educational differences in life expectancy between college or higher education and elementary or less education and between middle or high school and elementary or less education. These large contributions were not found in women. In addition, suicide in men was the most important contributor to the educational differentials in life expectancy between middle or high school and college or higher education and the second most important contributors to other educational differences. In women, cerebrovascular disease, suicide, transport accident, liver disease, and diabetes mellitus were the main contributors to life expectancy differences by educational levels. Among those, contributions by cerebrovascular disease and suicide were most important. The leading cancers in Korea, lung, stomach, and liver cancers, showed relatively greater mortality rates in low education groups than high education group and thus positively contributed to the educational differences in life expectancy among both men and women. However, prostate cancer and colorectal cancer among men and breast cancer and colorectal cancer among women contributed negatively to the differences in life expectancy by some educational levels. Ill-defined causes were also in accounting for educational life expectancy differences in both men and women.Figure 2 presents patterns of contributions by major causes of deaths to educational life expectancy differences by age groups. In men, suicide and liver disease contributed significantly to the educational differences in life expectancy in younger ages between 35 and 49, while major contributions by lung cancer and cerebrovascular disease were found among men aged 60 or over. Similar findings were observed among women. Suicide and liver disease showed important contributions in younger age groups such as ages 35–39 while in older age groups of women diabetes mellitus, cerebrovascular disease, and ischaemic heart disease contributed significantly to the education life expectancy differences. Meanwhile, the magnitude of contribution by ischaemic heart disease was small or negative in older age men.

## Discussion

Differences in life expectancy at age 25 between elementary or lower education (6 or less year schooling) and college or higher education (13 or more year schooling) in Korea were 16.23 years in men and 7.69 years in women. In Finland, the differences in life expectancy at age 30 between low education with 9 or less year schooling and high education with 13 or more year schooling were 6.96 years in men and 3.88 years in women in 1998–99, whereas the same educational differences in life expectancy at age 30 in Russia were 13.08 years in men and 10.21 years in women in 1998 [25,26]. The differences in life expectancy at age 25 between primary or lower education and university education in Lithuania were 16.75 years in men and 15.20 years in women in 2001 [27]. In Denmark, the educational differences in life expectancy at age 30 between primary or lower secondary education and tertiary education were 6.4 years in men and 4.7 years in women in 2011 [28]. Although it is hard to directly compare the magnitude of educational differences in life expectancy between countries due to the different educational categories and the study periods, results of this study suggest that the size of the educational differences in life expectancy seems to be relatively greater than that in northern European countries.

Younger age groups were more important contributors to the educational differentials in life expectancy between elementary or less education and other two higher educational groups, while older age groups were more important in explaining the difference between middle or high school education and college or higher education. This was generally true for both men and women in this study. This may mean that the dismal effects of poor socioeconomic environment would appear at younger ages among people with extreme social disadvantages (i.e., elementary or less education among young ages). In Korea where the enrollment rate in middle school increased from 42% in 1970 to over 90% in 1990 [20], only 0.4-13.0% of people aged 25–49 had elementary or less educational attainment [21]. People with elementary or less education aged less than 35 years may signify the extreme social exclusion in Korea. These young and socially marginalized population in Korea might have experienced neo-liberal structural reforms resulting in increases in unemployment rates, enhancement of labor flexibility, and rise of income inequality as well as lack of generous social safety net during the periods of the economic crisis in 1998 and the credit card crisis in 2003. The main contributing causes of deaths at those age groups to the educational differences in life expectancy were suicide and liver disease in both genders.

Korea has recorded the highest suicide rates among the Organization of Economic Co-operation and Development (OECD) member countries starting 2003 with upsurges during the Korea’s economic crisis in late 1990s and during the credit card crisis in 2003 [29,30]. Suicide is the most frequent cause of deaths in 20s’ and 30s’ men and women in Korea, although the elderly had the greater suicide rates than younger age groups [31]. Prior Korean studies showed that men and women aged 35–44 had greater educational differentials in suicide mortality in both relative and absolute terms than older age groups [30,32]. Results of this study as well as other prior studies suggest that social changes into harsher labor market environment might have had a greater impact on socioeconomically marginalized educational groups with younger ages who did not have sufficient resources and skills to overcome socioeconomic difficulties in late 1990s and early 2000s.

The main risk factors for liver disease are viral hepatitis and alcohol abuse [33,34]. According to the 2007 National Health and Nutrition Examination Survey of Korea by the Korea Centers for Disease Control and Prevention, the prevalence of the hepatitis B antigen positive among Koreans aged 19–49 is 2.1-4.3% and the prevalence of hazardous alcohol use is 44.5-45.8% [35]. Considering the relatively high rate of hepatitis B infection and alcohol abuse in Korea, social inequalities in hepatitis B viral infection and hazardous alcohol use might well have contributed to the significant part of the socioeconomic inequalities in liver disease [36-38].

Cerebrovascular disease and lung cancer in older age groups were the important causes of death in terms of differentials in life expectancy at age 25 especially between middle or high school and college or higher education. Cerebrovascular disease may be related to adverse childhood living conditions, along with liver disease, liver cancer and stomach cancer [39,40]. Poor socioeconomic environments and their inequitable distribution during and after Japanese colonial occupation (1910–1945) and the Korean War (1950–1953) might have had effects on the socioeconomic inequalities in mortalities from these causes. The prevalence of cigarette smoking, the main risk factor of lung cancer, reached over 50% before early 2000s with the highest rate being about 79% in 1980 among Korean men [41]. High smoking rates and high absolute differentials in smoking rates by educational level [42] might have contributed to the increase in mortality and mortality inequalities from lung cancer, especially in men. The percent contribution by cerebrovascular disease to the educational difference in life expectancy was greater among women than men while the percent contributions as well as absolute contributions (years) by lung cancer, stomach cancer, and liver cancer were greater in men than women. These results are similar to a previous study showing that, in women, the contribution of cerebrovascular disease was greater than that of cancer in southern and eastern European countries [1].

The biggest difference of the results in this study from findings in northern or western European countries is the size of the contribution by ischaemic heart disease to socioeconomic inequalities in mortality as indicated in prior Korean studies [32,43]. This study revealed that the contribution of ischemic heart disease was relatively small in Korea, accounting for 1-2% and 3-6% of total educational inequalities in life expectancy in men and women, respectively. Meanwhile, ischaemic heart disease was the most important contributor to total mortality inequalities in northern and western Europe [1,4]. However, mortality rates and absolute socioeconomic inequality in ischaemic heart disease are increasing rapidly in Korea [43]. Considering the secular trend of the westernized diet as risk factors for ischaemic heart disease in Korea [44], thorough monitoring on changes in socioeconomic inequalities of ischaemic heart disease is needed.

Our study has strengths and limitations. We presented age- and cause-specific contributions to the socioeconomic inequalities in life expectancy at age 25 using Arriaga’s decomposition method, while most previous studies showed only age-specific contributions and/or cause-specific contributions. Detailed quantification of age- and cause-specific contributions to socioeconomic inequalities in life expectancy allowed us to present varying age-specific contributions by each cause of deaths and to develop priority age groups and causes of deaths for each cause and age group. However, we used unlinked data with death certificate and census data which may produce a numerator-denominator bias [45]. A prior Korean study examined this issue [18]. When the educational level was categorized into three categories (elementary school or less, middle or high school graduate, college or higher), the percentage agreement between death certificate data and health survey data was 89.4% and the kappa value was 0.75 [18], which means the reliability level was substantial [46]. Thus, we believe that the numerator-denominator bias would be minimal.

## Conclusions

Educational differences in life expectancy were substantial in Korea. Liver disease and suicide were important contributors to the differences among younger age groups while cerebrovascular disease and lung cancer were important among older age groups. The age specific contributions for different causes of death to life expectancy inequalities by educational attainment varied with educational comparisons. Different age-specific distributions in educational levels due to remarkable improvement in education during the past decades may explain the findings as each educational attainment as SEP can have distinct meanings in the context and history in the Korean society. Exploring age- and cause-specific contributions to socioeconomic inequalities in life expectancy could allow us to better understand the nature of socioeconomic mortality inequalities and to specifically suggest priority areas for policy and intervention.

## Competing interests

The authors declare that they have no competing interests.

## Authors’ contributions

KJC participated in study design and drafted the manuscript. YHK conceived the original idea for the study and gave critical comments on the draft manuscript. HJC participated in study design and critical revision of the manuscript. SCY supervised study design, performed the statistical analysis and gave critical comments on the draft manuscript. All authors read and approved the final manuscript.

## Pre-publication history

The pre-publication history for this paper can be accessed here:

http://www.biomedcentral.com/1471-2458/14/560/prepub
